# Oculomotor Deficits after Chemotherapy in Childhood

**DOI:** 10.1371/journal.pone.0147703

**Published:** 2016-01-27

**Authors:** Einar-Jón Einarsson, Mitesh Patel, Hannes Petersen, Thomas Wiebe, Måns Magnusson, Christian Moëll, Per-Anders Fransson

**Affiliations:** 1 Department of Clinical Sciences, Lund University, Lund, Sweden; 2 Faculty of Medicine, University of Iceland, Reykjavik, Iceland; 3 School of Biosciences, University of East London, London, United Kingdom; 4 Division of Brain Sciences, Imperial College London, London, United Kingdom; 5 Department of Otorhinolaryngology, Landspitali University Hospital, Reykjavik, Iceland; 6 Department of Paediatrics, Skane University Hospital, Lund, Sweden; 7 Department of Otorhinolaryngology, Skane University Hospital, Lund, Sweden; Ospedale Pediatrico Bambino Gesu', ITALY

## Abstract

Advances in the diagnosis and treatment of pediatric malignancies have substantially increased the number of childhood cancer survivors. However, reports suggest that some of the chemotherapy agents used for treatment can cross the blood brain barrier which may lead to a host of neurological symptoms including oculomotor dysfunction. Whether chemotherapy at young age causes oculomotor dysfunction later in life is unknown. Oculomotor performance was assessed with traditional and novel methods in 23 adults (mean age 25.3 years, treatment age 10.2 years) treated with chemotherapy for a solid malignant tumor not affecting the central nervous system. Their results were compared to those from 25 healthy, age-matched controls (mean age 25.1 years). Correlation analysis was performed between the subjective symptoms reported by the chemotherapy treated subjects (CTS) and oculomotor performance. In CTS, the temporal control of the smooth pursuit velocity (velocity accuracy) was markedly poorer (p<0.001) and the saccades had disproportionally shorter amplitude than normal for the associated saccade peak velocity (main sequence) (p = 0.004), whereas smooth pursuit and saccade onset times were shorter (p = 0.004) in CTS compared with controls. The CTS treated before 12 years of age manifested more severe oculomotor deficits. CTS frequently reported subjective symptoms of visual disturbances (70%), unsteadiness, light-headedness and that things around them were spinning or moving (87%). Several subjective symptoms were significantly related to deficits in oculomotor performance. To conclude, chemotherapy in childhood or adolescence can result in severe oculomotor dysfunctions in adulthood. The revealed oculomotor dysfunctions were significantly related to the subjects’ self-perception of visual disturbances, dizziness, light-headedness and sensing unsteadiness. Assessments of oculomotor function may, thus, offer an objective method to track and rate the level of neurological complications following chemotherapy.

## Introduction

Recent advances in the diagnosis and treatment of pediatric malignancies have substantially increased the number of childhood cancer survivors. However, a number of the chemotherapeutic agents used in the treatment of childhood malignancies might cause neurological complications. The effects of chemotherapy are often not restricted to the target site. Although the brain itself is given some protection from systemic treatments by the blood-brain barrier, it has been recognized that many chemotherapeutic agents affect central brain function through direct and/or indirect mechanisms [1]. Such general damage to key CNS components might result in long-term decline to neurophysiological functions including motor control of the eyes, causing difficulties processing visual information [2].

In children, the somatosensory, visual and vestibular systems are in a state of development, which may make these systems more susceptible to neurotoxic effects from chemotherapy treatment. For example, when visual field deficits occur in both eyes and overlap, the corresponding part of the visual cortex may no longer be appropriately stimulated, which can influence the retinotopic organization of the visual cortex [3]. Hence, modified or absent stimulation of cortical areas in childhood might affect long-term development and quality of life, and thus, age at treatment may have a significant bearing on the severity of symptoms. However, most survivors of childhood cancer are not followed-up on a regular basis for the purpose of preventing, detecting and treating long-term neurophysiological deficits [4].

The long-term effect of chemotherapy treatment in childhood or adolescence on oculomotor functions is largely unknown to date. The oculomotor functions are vital for safe movement control and orientation as well as for many daily activities [5]. Assessments of oculomotor functions have therefore been regarded as providing one of the most important investigative windows for understanding numerous functions and dysfunctions of the human brain [6]. Saccadic eye movements are high-velocity, ballistic changes in eye position that bring an object of interest onto the fovea for sharp visualization. They can be triggered externally by a visual target suddenly appearing, known as a reflexive saccade. Smooth pursuit eye movements are tracking movements which ensure that the image of a moving object is maintained on the fovea [7]. Smooth pursuit eye movements may be triggered by following a target, known as foveal smooth pursuit [8]. Brain areas involved in the generation of saccadic and smooth pursuit eye movements include the frontal eye field, the supplementary eye field and the dorsolateral prefrontal cortex [9]. Although the neural pathways involved in smooth pursuits and saccades differ, one mutual requirement for correct eye movement control is a high level of accurate fine motor control.

The aim of this study was to evaluate reflexive saccade and foveal smooth pursuit eye movements in adult subjects treated with chemotherapy in childhood or adolescence for a solid malignant tumor not affecting the central nervous system. Another aim was investigate whether the severity of oculomotor deficits related to the age at treatment. Finally, we assessed whether the subjects experienced other effects often related to poor oculomotor control, e.g., dizziness, and determined if the intensity of these symptoms were related to oculomotor deficits.

## Materials and Methods

### Ethics Statement

The experiments were performed in accordance with the Helsinki declaration and approved by the Scientific Ethical Committee at Lund University, Sweden (number LU964-03) and by the Data Protection Authority (number LU-P6103), Sweden. A non obstat statement was obtained from the Scientific Ethical Committee, stating that no additional ethical approval was required to perform the investigations as part of clinical follow-up of the patient population. All participants or their guardians, provided written informed consent before the testing commenced.

### Subjects

The study included 48 subjects, 23 chemotherapy treated subjects (henceforth denoted CTS) and 25 healthy controls. The CTS were recruited from all adults who survived childhood cancer in the county of Skåne, Sweden, between 1980 and 2000. Twenty-three of approximately 750 adult survivors from the period, fulfilled the strict inclusion criteria: cancer diagnosed before the age of 18; treated for a solid malignant tumor not affecting CNS with chemotherapy agents and the treatment completed >5 years before this study. Subjects who had received cranial radiotherapy or surgery, which might affect the CNS, were excluded from participation. Subjects who fulfilled all criteria were contacted by a clinical administrator and offered to participate in the study, which they all did. The investigations were performed as part of a clinical follow-up of the patient population.

The 23 CTS participating in the study consisted of 11 females and 12 males of mean age 25.3 years (SD 6.7)). The mean age at diagnosis and treatment was 10.2 years (SD 5.1) and mean time between follow-up assessment and end of chemotherapy was 15.1 years (SD 5.6). Treatment details are presented in Table 1. To determine whether the developmental state at treatment influenced oculomotor function, the CTS group was also divided into two age subgroups. One subgroup (CTS_Young), included all CTS that received treatment before 12 years of age, consisted of 14 subjects (8 women, mean age 23.1 years (SD 7.1)) with mean age at treatment of 7.1 years (SD 3.4). The other subgroup (CTS_Old), included all CTS that received treatment at 12 years of age or older, consisted of 9 subjects (3 women, mean age 28.0 years (SD 5.4)) with mean age at treatment of 15.3 years (SD 1.9). Statistical and manual analyses verified that the two age subgroups had not systematically received different chemotherapy treatments.

**Table 1:** Subject characteristics, diagnosis and chemotherapy details.

Subject	Diagnosis	Gender	Age at treatment (years)	Age when assessed (years)	Chemotherapy treatment agents*
1	Sacrococcygeal teratoma	Female	0.1	23.4	Ble, Cis, Eto
2	Hepatoblastoma	Female	2.5	15.9	Adr, Cis
3	Embryonal teratoma	Female	2.5	17.7	Ble, Cis, Eto
4	Ewing sarcoma	Male	2.9	16.4	Act, Adr, Eto, Ifo, Vin
5	Osteosarcoma	Female	6.1	17.5	Adr, Cis, Met
6	Osteosarcoma	Female	8.4	15.5	Adr, Cis, Ifo, Met
7	Ewing sarcoma	Female	8.6	30.0	Act, Adr, Ble, Cyc, Met, Vin
8	Ewing sarcoma	Male	8.7	27.7	Act, Adr, Ble, Cyc, Met, Vin
9	Neuroblastoma	Male	8.9	21.4	Car, Cis, Cyc, Eto, Mel, Vin
10	Immature teratoma	Female	9.1	18.5	Ble, Cis, Eto
11	Ewing sarcoma	Male	9.6	30.3	Act, Adr, Ble, Cyc, Met, Vin
12	Osteosarcoma	Male	9.9	27.6	Act, Adr, Ble, Cis, Cyc, Met
13	Immature teratoma	Female	10.3	35.8	Act, Adr, Cyc, Vin
14	Ewing sarcoma	Male	10.7	33.1	Act, Adr, Ble, Cis, Cyc, Met, Vin
15	Ewing sarcoma	Female	12.1	18.4	Act, Adr, Cyc, Eto, Ifo, Vin
16	Osteosarcoma	Female	12.6	27.4	Adr, Cis, Met
17	Osteosarcoma	Female	14.3	33.9	Act, Adr, Ble, Cis, Cyc, Met
18	Ewing sarcoma	Male	15.5	35.4	Act, Adr, Ble, Cyc, Met, Vin
19	Ewing sarcoma	Male	15.7	24.0	Adr, Cis, Ifo, Vin
20	Immature teratoma	Male	16.5	27.8	Ble, Cis, Eto
21	Ewing sarcoma	Male	16.8	23.7	Act, Adr, Cis, Cyc, Eto, Ifo, Vin
22	Osteosarcoma	Male	16.9	30.9	Adr, Cis, Met
23	Osteosarcoma	Male	17.0	30.4	Adr, Cis, Eto, Ifo, Met

* Act: Actinomycin-D; Adr: Adriamycin; Ble: Bleomycin; Car: Carboplatin; Cis: Cisplatin; Cyc: Cyclophosphamide; Eto: Etoposide; Ifo: Ifosfamide; Mel: Melphalan; Met: Methotrexate; Vin: Vincristine.

The age and gender-matched control group consisted of 25 healthy participants (13 women, mean age 25.1 years (SD 4.6). All subjects included in the study, both controls and CTS, had normal or corrected to normal visual acuity using glasses or contact lenses.

### Procedure & assessments

**Fig 1:**
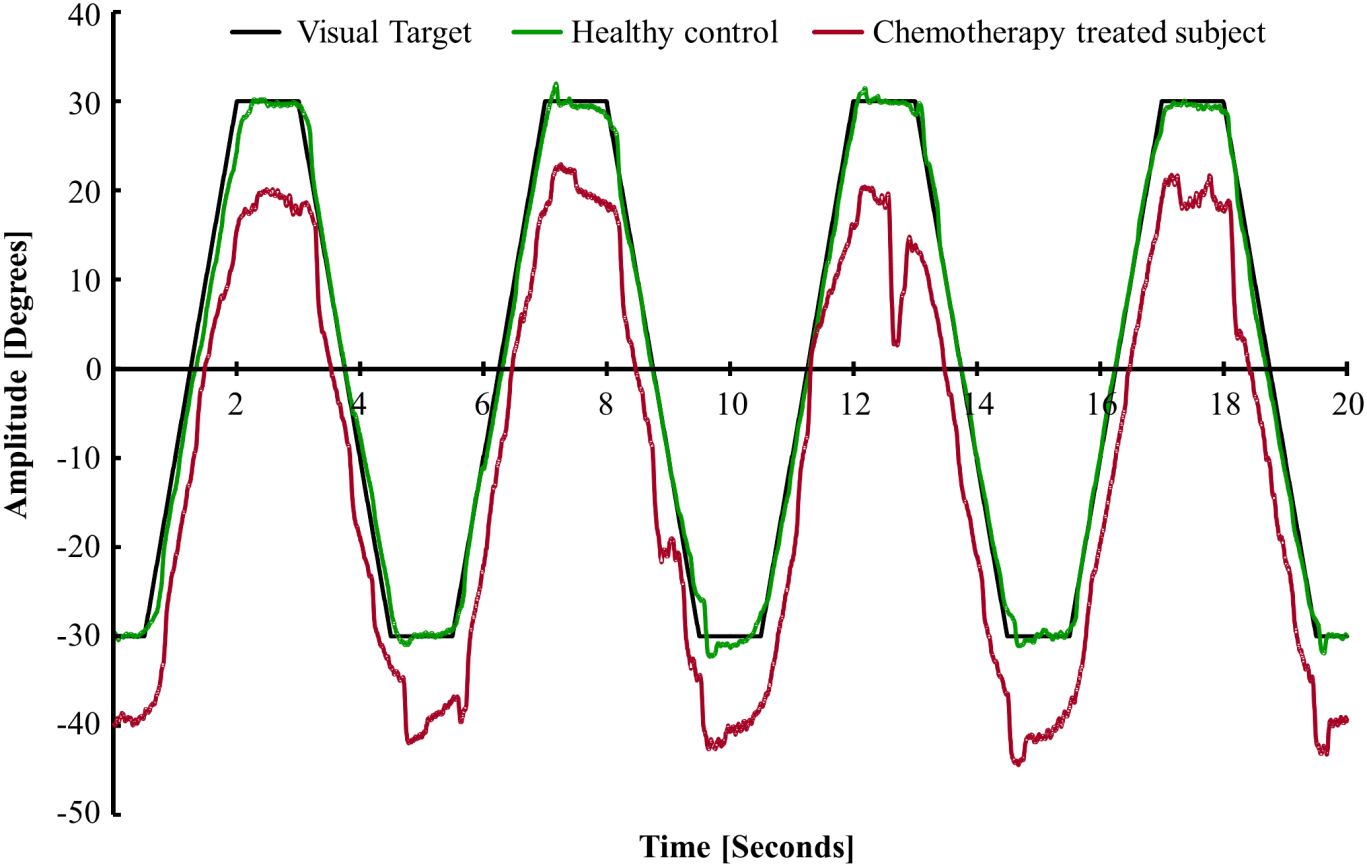
Illustration of smooth pursuit eye movements made by a CTS and a healthy subject. In the recording section presented, the healthy control (green line) and CTS (red line) subjects aimed to focus on a visual target (black) moving at 40°/s. For better graphical presentation, the recording from the CTS were moved -10 degrees downwards. The CTS subject could poorly maintain for longer periods a stable eye velocity matching the visual target movements.

The visual target used in the oculomotor tests was a circular red dot with a diameter of 3mm, projected about 1.3m in front of the subjects using a diode laser. Eye movements were recorded by a custom-made electronystagmography (ENG) using a bipolar recording technique. The eye movement data were initially filtered by appropriate analogue filters to eliminate aliasing and environmental noise and then processed by a custom-made device designed to reduce DC drifts before the signal was sampled at 200 Hz. Prior to each test, a calibration procedure was performed to ensure that the ENG signals corresponded correctly to eye movement in the horizontal direction with an error less than 1 degree within the range of ±30 degree amplitude. See typical recording examples of a CTS subject and a healthy subject in Fig 1. A customized software program Vestcon^™^ controlled the visual target projection, sampled and analyzed the ENG data. All subjects in the study were assessed using the same smooth pursuit and saccade test sequences.

#### Smooth pursuit eye movement recordings

Each subject was tested in a completely dark room and seated in an inclined chair directly in front of a large black canvas screen. Subjects were then instructed to fixate on a red target projected onto the screen and follow its horizontal movement as accurately as possible without turning their head or moving their eyes before the target had moved. Inappropriate head movements were prevented by a custom-made headrest. The smooth pursuit target moved horizontally with a constant velocity from side to side, with a range of ±30° of the visual field, i.e. a distance of 60° between (+) 30° to the right and (-) 30° to the left. The velocity of visual target followed the fixed sequence: 10, 20, 30, 40, 40, 30, 20, 10°/s, the target movement repeated 4 times at each sequence step with two movements in each direction. When the visual target reached the maximum amplitude, i.e., ±30° either to the right or left, the position was maintained for 1 second before the next movement commenced in the opposite direction.

#### Saccadic eye movement recordings

The conditions and calibration before saccade testing were identical to smooth pursuit recordings, as were the test instructions. The visual target jumped stepwise horizontally according to the following sequence of amplitudes: ±10, ±20, ±30°, yielding target movements of 20, 40 and 60° amplitude respectively. The visual target appeared for 1.5s at each position. The saccades were tested 10 times at each of the amplitudes, five times for movements in each direction.

### Oculomotor data analysis

The oculomotor analyses performed are described in detail elsewhere [10, 11], though in brief the following oculomotor properties were analyzed:

#### Smooth pursuit parameters

The “latency”, defined as the mean time from the start of target movement till the eye velocity exceeded 5°/s. The “gain”, defined as the quotient between mean eye movement velocity and the target velocity for the time periods where the eye movements fulfilled to the criteria of being proper smooth pursuits. The “velocity accuracy”, defined the temporal stability of the smooth pursuit control function by calculating the percentage of time the eye movement velocity was within the boundaries of less than 20% absolute error from the target velocity.

#### Saccade parameters

The “latency”, defined as the mean time from the start of target movement till the eye velocity exceeded 80°/s. The “peak saccade velocity”, defined by identifying the 25ms period with the highest saccade velocity during the entire saccade movement and then calculate the average velocity during this 25ms period. The “amplitude accuracy”, defined as the quotient in percent between the amplitude of the largest saccade made during the saccade movement divided by the visual target movement. The “main sequence”, defined the relationship between peak saccade velocity and saccade amplitude. A main sequence value higher than normal means that the saccades had disproportionally shorter amplitude than normal for the recorded saccade peak velocity [12].

### Subjective symptoms questionnaire

All CTS filled out a version in Swedish of the questionnaire (The Vertigo Symptom Scale (VSS) developed by Yardley [13]), as part of rating the level of experienced handicap. The questionnaire measures symptoms related to dizziness, imbalance and vision.

### Statistical Analysis

The data from rightward and leftward smooth pursuit and saccadic eye movements were pooled together in the analysis since no systematic directional differences were discernable in the statistical analysis [10]. The oculomotor parameters were analyzed using repeated measures GLM ANOVA. For smooth pursuit parameters the main factors analyzed were: ‘Chemotherapy’ (CTS or Controls; d.f. 1) and ‘Target Velocity’ (10°/s, 20°/s, 30°/s or 40°/s: d.f. 3). For saccade parameters the main factors analyzed were: ‘Chemotherapy’ (CTS or Controls; d.f. 1) and ‘Target movement Amplitude’ (20°, 40° or 60°: d.f. 2).

The Mann-Whitney test was used for pair-wise post hoc comparisons. Spearman’s Rank correlations were performed between CTS’ questionnaire scores and recorded oculomotor parameters to determine whether the level of symptoms experienced were related to recorded levels of deficits in oculomotor functions. The CTS age subgroups were regarded too small for correlation analysis. In the GLM ANOVA and correlation analyses, a p<0.05 was considered significant, whereas in the Mann-Whitney post hoc tests a p<0.017 was considered significant adhering to Bonferroni. Trends (p<0.1) are marked in the figures and tables. Non-parametric statistics were used since not all data sets analyzed were normally distributed before or after logarithmic transformation.

## Results

### Smooth pursuit eye movements

The CTS were commonly able to make smooth pursuits of mean velocity fairly close to the target velocity, see Fig 1. However, the CTS had poor control of the temporal accuracy of the eye velocity.

#### Role of chemotherapy treatment

The GLM ANOVA revealed that the CTS had 17% (p<0.001) lower velocity accuracy compared with controls, see Table 2. The velocity accuracy in CTS_Young was 23% (p<0.001) lower compared with controls, whereas 10% (p = 0.027) lower in CTS_Old compared with controls. Moreover, the CTS_Young had 10% (p = 0.025) lower velocity accuracy compared with CTS_Old.

**Table 2:** Effects of chemotherapy and visual target velocity on smooth pursuit parameters.

Smooth pursuit parameters*	Chemotherapy**	Target velocity**	Chemotherapy x Target velocity**
CTS vs healthy controls			
Velocity accuracy	< 0.001 [25.1]	< 0.001 [129.0]	0.382 [0.8]
Gain	0.120 [2.5]	< 0.001 [34.0]	0.066 [3.5]
CTS_Young vs healthy controls			
Velocity accuracy	< 0.001 [31.2]	< 0.001 [91.6]	0.104 [2.7]
Gain	0.042 [4.3]	< 0.001 [36.4]	0.099 [2.8]
CTS_Old vs healthy controls			
Velocity accuracy	0.027 [5.1]	< 0.001] 90.3]	0.471 [0.5]
Gain	0.854 [0.0]	0.001 [11.7]	0.089 [3.0]
CTS_Young vs CTS_Old			
Velocity accuracy	0.025 [5.4]	<0.001 [46.0]	0.141 [2.2]
Gain	0.151 [2.1]	<0.001 [15.0]	0.111 [2.7]

* Repeated measures GLM ANOVA analysis of how the smooth pursuit parameters were affected by main factors “Chemotherapy” and “Target velocity” alone and by the main factor interaction denoted as “Chemotherapy x Target velocity”.

** The statistical F-values are presented within the squared parentheses.

Gains were not significantly different between CTS and controls. However, gain was 3% (p = 0.042) lower in the CTS_Young group compared with controls.

#### Role of target velocity

The GLM ANOVA revealed that the velocity accuracy gradually increased by on average 7% (p<0.001) from the lowest values at 10°/s target velocities till 40°/s velocities, for all group comparisons presented in Table 2.

The gain gradually decreased by about 4% (p≤0.001) from the highest gain at 10°/s target velocities to 40°/s velocities for all group comparisons.

#### Role of chemotherapy treatment interacting with target velocity

The GLM ANOVA showed no significant interacting effects on smooth pursuit parameters between receiving chemotherapy and the target velocity, see Table 2.

#### Post hoc analysis of smooth pursuit parameters

**Fig 2:**
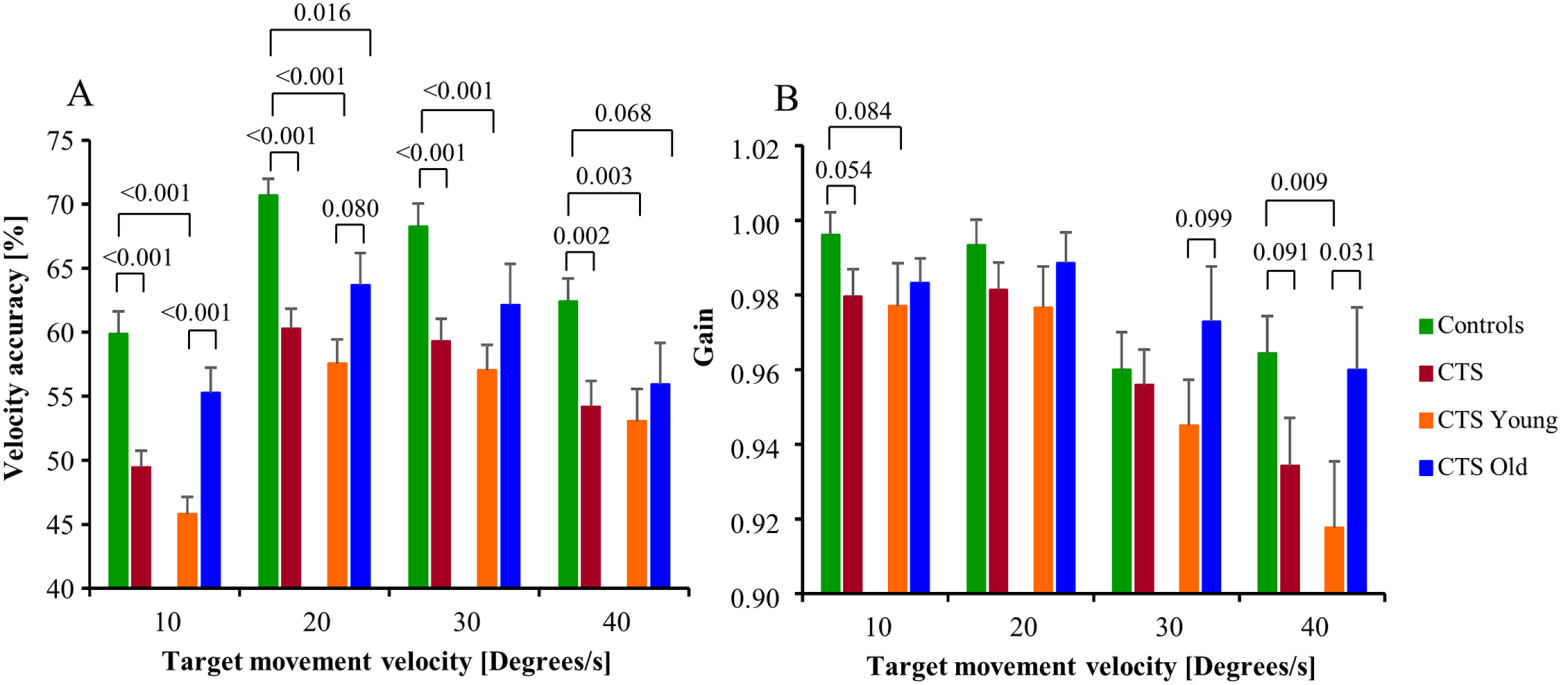
Smooth pursuit parameter values. (A) Velocity accuracy, representing the percentage of time the smooth pursuit velocity differed less than 20% from the visual target velocity. (B) Gain, where a value below 1.0 represents that the smooth pursuit velocity was below the visual target velocity. The bars represent the mean group values and the whiskers the SEM values. P-values to the level of trends <0.1 are presented in the figures.

The post-hoc Mann-Whitney analysis revealed that the velocity accuracy was on average 17% (p≤0.002) lower in CTS at all target velocities compared with controls; see Fig 2A. In the CTS_Young group, the velocity accuracy was on average 23% (p≤0.003) lower for all target velocities compared with controls, whereas in CTS_Old 11% (p = 0.016) lower for the 20°/s targets compared with controls. Moreover, the velocity accuracy was 17% (p<0.001) lower in CTS_Young compared with CTS_Old for the 10°/s targets.

Gain was 5% (p = 0.009) lower in CTS_Young compared with controls for 40°/s targets, see Fig 2B.

### Saccade eye movements

**Fig 3:**
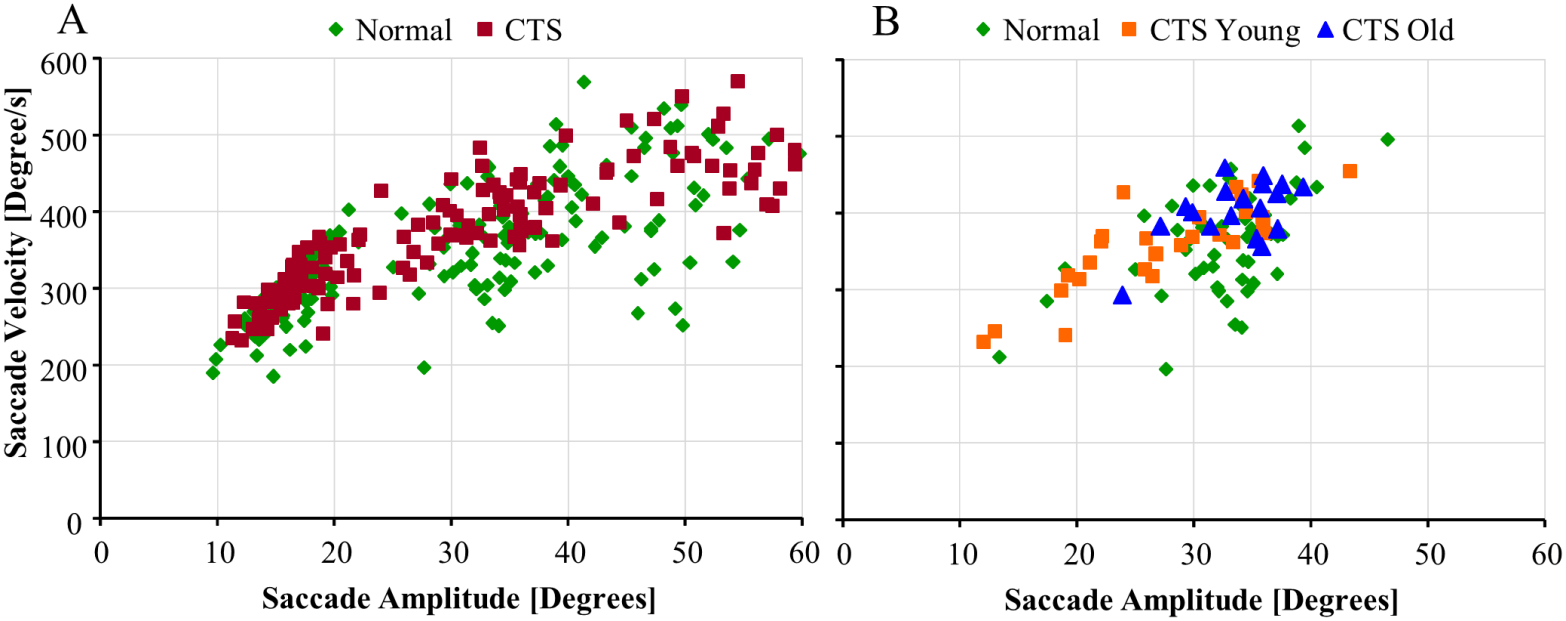
Main sequence velocity vs. amplitude diagram for CTS and controls. (A) The relationship between peak velocity and amplitude is close to linear up to about 20° amplitude, to thereafter become increasingly non-linear with increasing saccade amplitude. The CTS regularly had higher peak velocity than controls at similar amplitudes. (B) The saccade movements to 40° visual target movement display that in CTS_Young the peak velocity relates to shorter distance moved than in controls or CTS_OLD.

Typically, the relationship between peak saccade velocity and saccade amplitude is linear up to about 20° amplitude to thereafter become increasingly non-linear with increasing saccade amplitude [10, 12], see Fig 3A. However, in saccades made by CTS_Young, the saccade amplitudes produced were disproportionally shorter than normal for the saccade peak velocity used, see Fig 3B.

#### Role of chemotherapy treatment

The GLM ANOVA revealed that the peak saccade velocity was not significantly different in CTS compared with controls, see Table 3. However, the peak velocity was higher by 10% (p = 0.023) in CTS_Old compared with controls and higher by 7% (p = 0.040) in CTS_Old compared with CTS_Young.

**Table 3:** Effects of chemotherapy and visual target movement amplitude on saccade parameters.

Saccade parameters*	Chemotherapy	Target amplitude	Chemotherapy x Target amplitude
CTS vs healthy controls			
Peak velocity	0.059 [3.6]	< 0.001 [528.7]	0.037 [4.5]
Amplitude accuracy	0.587 [0.3]	0.020 [5.6]	0.016 [6.0]
Main Sequence	0.004 [8.7]	< 0.001 [1526.4]	0.015 [6.2]
CTS_Young vs healthy controls			
Peak velocity	0.410 [0.7]	< 0.001 [411.8]	0.064 [3.5]
Amplitude accuracy	0.125 [2.4]	0.002 [9.8]	0.003 [9.6]
Main Sequence	0.005 [8.4]	< 0.001 [1083.9]	0.001 [11.0]
CTS_Old vs healthy controls			
Peak velocity	0.023 [5.4]	< 0.001 [303.7]	0.032 [4.8]
Amplitude accuracy	0.241 [1.4]	0.136 [2.3]	0.035 [4.7]
Main Sequence	0.176 [1.9]	< 0.001 [1099.6]	0.081 [3.2]
CTS_Young vs CTS_Old			
Peak velocity	0.040 [4.5]	< 0.001 [261.3]	0.085 [3.1]
Amplitude accuracy	0.038 [4.6]	0.220 [1.6]	0.036 [4.7]
Main Sequence	0.278 [1.2]	< 0.001 [655.3]	0.016 [6.3]

* Repeated measures GLM ANOVA analysis of how the saccade parameters were influenced by the main factors “Chemotherapy” and “Target amplitude” alone and by the main factor interaction denoted as “Chemotherapy x Target amplitude”.

The amplitude accuracy was not significantly different between CTS and controls. However, the amplitude accuracy was higher by 12% (p = 0.038) in CTS_Old compared with CTS_Young.

The main sequence values were higher by 8% (p = 0.004) in CTS compared with controls and higher by 8% (p = 0.005) in CTS_Young compared with controls.

#### Role of target movement amplitude

The GLM ANOVA revealed that the peak velocity increased by on average 47% (p<0.001) from a 20° target movement to a 60° target movement in all group compositions, see Table 3.

The amplitude accuracy was on average 6% (p≤0.020) higher at 20° target movements than at 60° movements for the CTS vs control and CTS_Young vs control group comparisons.

The main sequence values were reduced by on average 48% (p<0.001) from a 20° target movement to a 60° target movement in all group compositions.

#### Role of chemotherapy treatment interacting with target movement amplitude

The CTS had higher peak velocities for the larger target movements compared to controls (p = 0.037), i.e., 4% faster at 20° target movements but 9% faster at 60° movements, see Table 3. Moreover, the CTS_Old had higher peak velocities for the larger target movements than controls (p = 0.032), i.e., 6% faster at 20° movements but 12% faster at 60° movements.

The CTS had higher amplitude accuracy for the larger target movements compared to controls (p = 0.016), from about identical values for both groups at 20° movements to 4% accuracy increase at 60° movements for CTS. On the other hand, CTS_Young had lower amplitude accuracy for the smaller target movements than controls (p = 0.003), i.e., 2% lower at 20° movements but close to identical at 60° movements. The CTS_Old had significantly higher amplitude accuracy for larger target movements compared to controls (p = 0.035), i.e., close to identical at 20° movements but 12% higher at 60° movements. Furthermore, the CTS_Young had lower amplitude accuracy for the larger target movements than CTS_Old (P = 0.036), i.e., 1% lower at 20° movements but 12% lower at 60° movements.

The CTS had higher main sequence values for larger target movements than controls (p = 0.015), i.e., 5% larger at 20° movements but 7% larger at 60° movements. Furthermore, the CTS_Young had higher main sequence values for larger target movements than controls (p = 0.001), i.e., 5% larger at 20° movements but 10% larger at 60° movements, and higher main sequence values for larger target movements than CTS_Old (p = 0.016), i.e., 1% lower at 20° movements but 7% larger at 60° movements.

#### Post hoc analysis of saccade parameters

**Fig 4:**
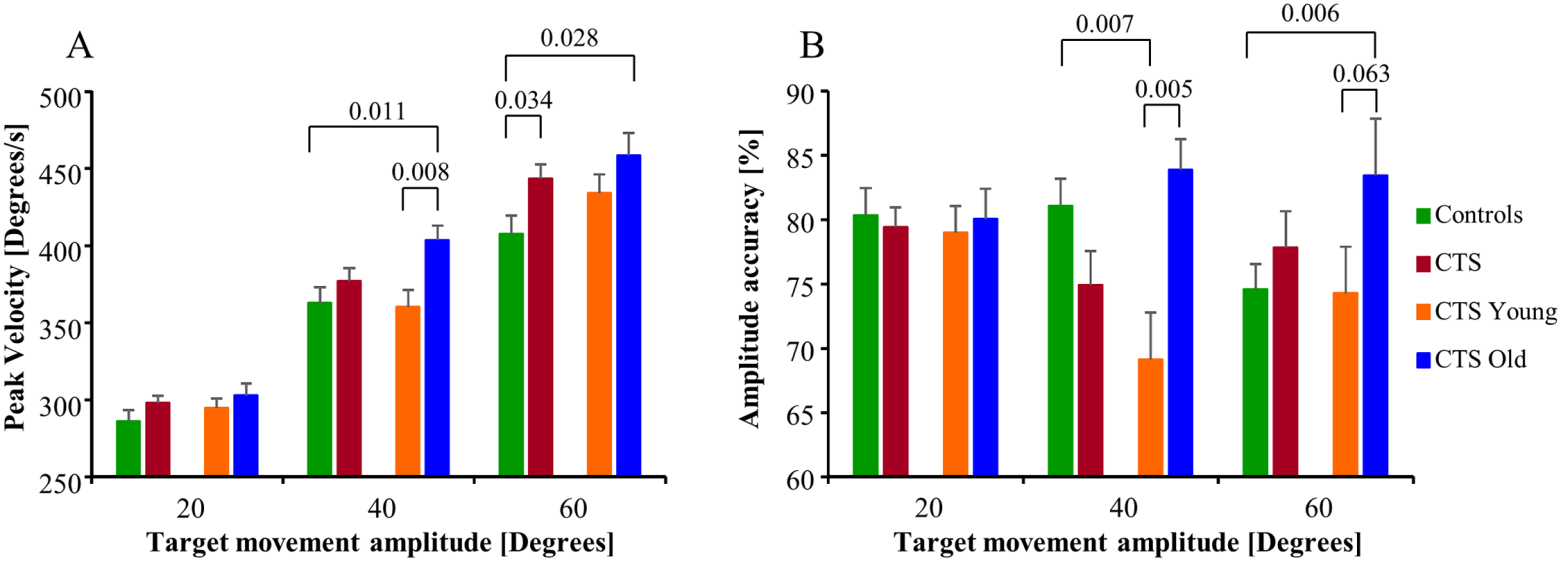
Saccade parameter values. (A) Saccade peak velocity. (B) Amplitude accuracy, where a value below 100% represent that the saccade amplitude was smaller than the visual target jump (hypometric). The bars represent the mean group values and the whiskers the SEM values.

The peak velocity was 11% (p = 0.011) higher in CTS_Old compared with controls for 40° target movements, and 12% (p = 0.008) higher in CTS_Old compared with CTS_Young for 40° targets, see Fig 4A.

The amplitude accuracy was 15% (p = 0.007) lower in CTS_Young compared with controls for 40° targets, whereas 12% (p = 0.005) higher in CTS_Old compared with controls for 60° targets, see Fig 4B. The amplitude accuracy was 21% (p = 0.005) lower in CTS_Young compared with CTS_Old for 40° targets.

**Fig 5:**
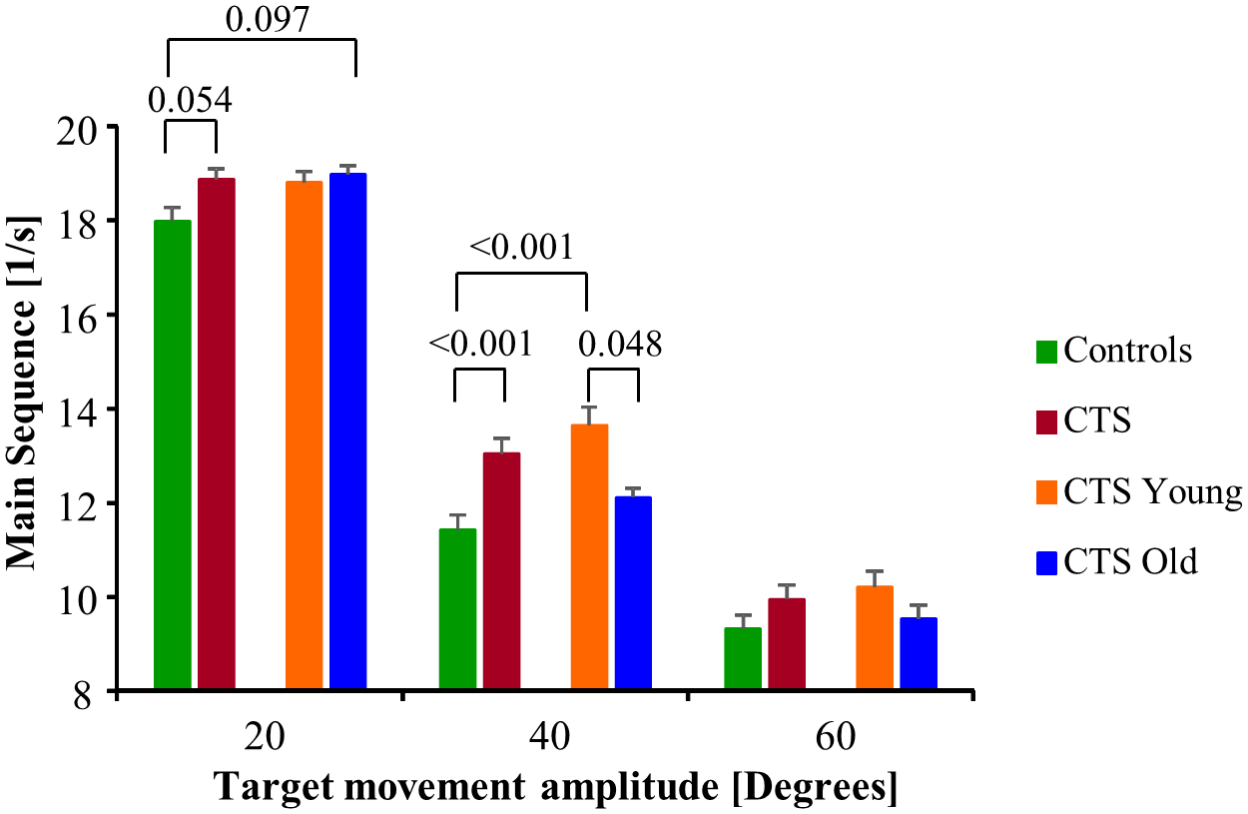
Main sequence parameter values. The main sequence values (mean and SEM values) illustrate the relationship between the saccade’s peak velocity and amplitude. A higher main sequence value than normal suggests that the saccades produced moved disproportionally shorter distance than normal for the saccade peak velocity.

The main sequence values were 14% (p<0.001) larger for CTS compared with controls for 40° targets and 20% (p<0.001) larger for CTS_Young compared with controls for 40° targets, see Fig 5.

#### Smooth pursuit and saccade latencies

**Fig 6:**
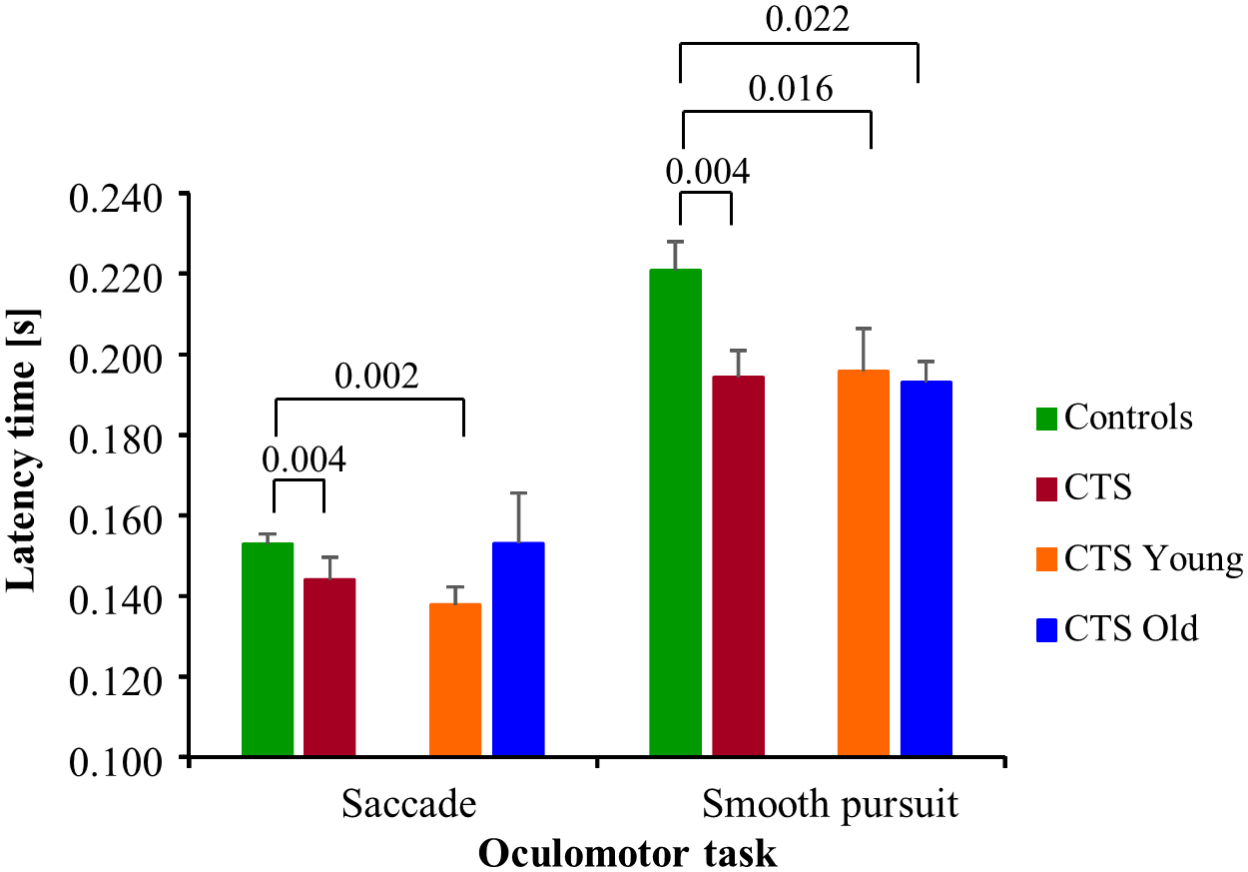
Smooth pursuit and saccade latency parameter values. Initiation response latencies (mean and SEM values) when performing the saccade and smooth pursuit oculomotor tasks.

Both the smooth pursuit and saccade latencies were shorter in CTS compared with controls, by 14% (p = 0.004) and 6% (p = 0.004) respectively, see Fig 6. Similarly, the smooth pursuit and saccade latencies were shorter in CTS_Young compared with controls, by 13% (p = 0.016) and 11% (p = 0.002) respectively.

### Analysis of the questionnaire

The questionnaire revealed that CTS experienced a range of symptoms, see Table 4. The most common symptoms were visual disturbances (i.e. blurred, flickering, blind spots in the visual field) (in 70% of CTS) and headache (96%). Most CTS experienced unsteadiness, light-headedness or the feeling that things around them were spinning or moving (87%). Other frequent symptoms were nausea (61%), tension or soreness in the muscles (61%), poor concentration (52%) and feelings of fainting (48%).

**Table 4:** VSS questionnaire answers from the CTS subjects (n = 23) to the question: “How often during the last 12 months have you felt the following symptoms:”

VSS questions	Between 1–12 times per year*	More than once a month*	In total*
1. Heart/chest pain?	6	4	10
2. Hot or cold spells?	5	3	8
3. Falling over?	2	0	2
4. Nausea, feeling sick?	14	0	14
5. Tense/sore muscles?	12	2	14
6. Trembling/shivering?	4	1	5
7. Pressure in the ears?	6	2	8
8. Heart pounding?	8	2	10
9. Vomiting?	3	0	3
10. Arms/legs feel heavy?	7	1	8
11. Visual disturbance?	10	6	16
12. Headache?	15	7	22
13. Unable to stand, walk?	2	0	2
14. Breathing difficulties?	3	1	4
15. Poor concentration?	10	2	12
16. Tingling, pricking?	3	3	6
17. Lower back pain?	6	4	10
18. Excessive sweating?	2	2	4
19. Feeling faint?	10	1	11
20. Things spinning/moving, lasting:			
- Less than 2 min	15	1	16
- 2 to 60 min	2	0	2
- More than 1 hour	0	1	1
- Whole day	0	0	0
**- In total**	**17**	**2**	**19**
21. Light-headedness/giddiness, lasting:			
- Less than 2 min	11	2	13
- 2 to 60 min	3	1	4
- More than 1 hour	3	0	3
- Whole day	0	0	0
**- In total**	**17**	**3**	**20**
22. Unsteadiness, lasting:			
- Less than 2 min	6	1	7
- 2 to 60 min	1	0	1
- More than 1 hour	0	0	0
- Whole day	0	0	0
**- In total**	**7**	**1**	**8**

* Subjects not grouped for the question in the table had not experienced the symptoms described during the period.

The correlation analyses revealed strong relationships between CTS’ self-reported symptoms and smooth pursuit performance, see Table 5. For the 16 questionnaire questions where the answer distribution allowed performing a correlation analysis, 26 significant correlations suggested a relationship between decreased smooth pursuit performance and increased frequency of subjective symptoms of, e.g., feeling headache, dizziness, unsteadiness and visual disturbances.

**Table 5:** Significant correlations and trends between questionnaire answers, smooth pursuit and latency parameters.

	Velocity accuracy*				Gain*				Latency*
Q.nr.	10°/s	20°/s	30°/s	40°/s	10°/s	20°/s	30°/s	40°/s	
1	<0.001 [-.545]	0.024 [-.333]			0.069 [-.271]			0.058 [-.282]	
2	0.082 [-.259]	0.005 [-.404]							
4		0.025 [-.329]							
5	0.018 [-.348]								
7	0.005 [-.404]					0.006 [.401]	0.011 [.371]		0.009 [.379]
8	0.040 [-.303]								
10	0.005 [-.407]								
11	0.003 [-.427]	0.099 [-.247]		0.083 [-.259]			0.066 [-.274]	0.020 [-.341]	
12		0.028 [-.324]	0.064 [-.276]	0.046 [-.296]		0.013 [-.365]	0.007 [-.394]	0.002 [-.439]	
15				0.092 [-.251]					
16	0.002 [-.449]	0.019 [-.345]						0.021 [-.339]	
17	0.006 [-.396]								0.030 [.330]
19	0.003 [-.431]				0.035 [-.311]			0.053 [-.288]	
20a	0.002 [-.451]	0.082 [-.259]							
21a	<0.001 [-.508]	0.079 [-.261]						0.068 [-.272]	
22a	0.005 [-.411]	0.009 [-.379]	0.013 [-.363]						

* Correlations were performed when the symptoms were recognized by more than 5 subjects (i.e., by more than 22% of the subjects in the CTS group). The correlation R-values are presented within the squared brackets. A negative R-value represents that a poorer oculomotor function was related to more frequent subjective symptoms. The questions are spelled out in Table 4.

The correlations between saccade parameters and the CTS’ self-reported symptoms revealed 10 significant relationships, though in 8 cases these related to autonomic symptoms. However, higher main sequence values at 20° correlated with more frequent problems with visual disturbances (R = 0.319, p = 0.031), whereas longer saccade latency correlated with increased feelings of unsteadiness (R = 0.320, p = 0.032).

## Discussion

The main objective of cancer treatment is to ensure patient survival. However, certain chemotherapy treatments in childhood may influence quality of life in survivors. Our findings revealed that adults who received chemotherapy treatment in childhood or adolescence experienced severe oculomotor deficits later in life, manifested as poorer temporal control of smooth pursuit velocities at all tested target velocities (velocity accuracy) (p<0.001) and by producing saccades with disproportionally shorter amplitude than normal for the saccade peak velocity (main sequence) (p = 0.004). Both findings suggest impaired oculomotor control, possibly related to poorer neuromotor drive efficiency or to temporal instabilities in the oculomotor control systems. Moreover, those treated with chemotherapy at younger age had more severe deficits in oculomotor performance than those treated at older age. Finally, the CTS commonly experienced symptoms of headache, dizziness, unsteadiness and visual disturbances, which related to the oculomotor deficits.

### The route of potential impairment

Our results suggest that the control networks operating smooth pursuits and saccades are susceptible, especially in childhood, to some of the chemotherapy agents used for treatment. The predominant drive for smooth pursuit initiation is retinal slip (i.e. moving target velocity on the retina), although target position and acceleration also contribute [14]. After initiation, afferent visual feedback as well as efference copies of the motor commands are used for pursuit maintenance [15], i.e. the sensory velocity information from the retina must be continually updated and processed for correct smooth pursuits which requires high levels of continuous, accurate and fast fine motor control.

However, the limited capacity of the visual system means that a very quick movement of the target cannot be tracked in real-time, and in these cases a saccade is generated. Once the selected target region is defined, saccadic landing positions can be determined by the spatial pooling of visual signals [16]. Hence, visual feedback and accurate motor control play less significant roles in reflexive saccades than foveal smooth pursuits. This could be one reason why we observed a generalized deficit in the smooth pursuit velocity accuracy parameter in CTS, see fig 1, whereas the saccadic deficits found in CTS were more subtle, affecting saccades within larger amplitude ranges rarely investigated.

Both the latency of smooth pursuit and saccades were significantly shorter in CTS than for healthy subjects. The preparation of pursuit and saccades starts at the same time, initiated by the same shift of attention, with the execution of each movement by a separate control system [17]. Furthermore, the same neurons in the superior colliculus respond prior to the selection of the target, regardless of whether the movement will be carried out as a smooth pursuit or saccade [18]. One possible explanation for the shorter saccade and smooth pursuit latencies in CTS could thus be that this is a functional compensation made to keep the object of interest on the fovea when smooth pursuit eye movement functions are impaired, i.e., through faster initiation of corrective actions.

The pattern of deficit found with the novel smooth pursuit velocity accuracy parameter largely mirrors the one found with the traditional smooth pursuit gain parameter, but the sensitivity of the novel method was higher and correlated to the subjects’ self-reported symptoms better [10, 11]. Hence, one of the first signs of impaired oculomotor function might be an inability to maintain an accurate and stable control of smooth pursuit movements in temporal space within acceptable boundaries over longer periods of time [19]. The parameter’s high sensitivity in detecting chemotherapy-related impairments suggests that temporal analysis of smooth pursuit eye movements may reveal oculomotor deficits in earlier stages than traditional methods.

### Age at time of treatment and time after chemotherapy treatment effects

The developmental state of being a child or adolescent at the time of treatment was an important factor in predicting the severity of deficits in adulthood. Subjects treated at younger (<12 years) age with chemotherapy had more severe deficits in oculomotor control than subjects treated in adolescence. Those treated at younger age also reported more subjective problems and more frequent symptoms.

Neurotoxic signs and symptoms tend to disappear over time as a result of adaptation and plasticity compensation processes [20, 21]. However, the CTS experienced severe deficits even on average 15 years after the end of treatment, which suggests a poor ability to recover from damage caused by chemotherapy in childhood. A possible reason for this could be that in young children, the somatosensory, visual and vestibular systems and associated CNS functions are still in a state of development, which may make these systems more susceptible to neurotoxic effects disrupting the developmental processes. However, large scale studies are required to determine why age at treatment is of such evident importance. Moreover, the findings presented highlight the importance of monitoring the health status among CTS for at least 15 years after treatment but also suggest that this patient population should be monitored with pre- and early post-treatment screening programs as well as with consecutive screening throughout childhood to detect immediate and progressive late effects from the treatments received. Here, oculomotor assessments may provide a window for observing neurological impairments and recovery in the early phases of chemotherapy in childhood. As a result of these findings, both Skåne University Hospital, Lund, Sweden and Landspitali University Hospital, Reykjavik, Iceland have instigated discussions about how to design and implement appropriate screening procedures and rehabilitation methods of this patient population to address late effects such as oculomotor deficits.

### General drug effects

As the CTS received a large variety of dosages and combinations of chemotherapeutic agents, the study material was regarded insufficient to reliably determine and formally state whether specific agents or dosages were directly associated with causing oculomotor impairments. However, correlation analysis revealed significant relationships between increasing oculomotor deficits and cumulative dosages of the three chemotherapy agents; cisplatin, methotrexate and ifosfamide.

Chemotherapy has the potential to cause general CNS damage, which includes the blood-brain barrier [22, 23]. Clinically relevant concentrations of 1,3-bis(2-chlorthyl)-1-nitrosoura (BCNU) and cisplatin are more toxic to CNS progenitor cells and oligodendrocytes than to cancer cell lines [22]. Furthermore, therapeutic levels of 5-fluorouracil (5-FU) is associated with progressive delayed damage to myelin [23]. Thus, there are potential risks with using concoctions of agents, which is common when treating child cancer patients. Agents which would otherwise be prevented from damaging the brain by the blood-brain barrier when administered independently may have this means if an agent administered in conjunction is toxic to the barrier.

Particularly relevant for oculomotor performance is that vincristine can induce neuropathy to cranial nerves and especially of the oculomotor nerves [24, 25]. Vincristine and methotrexate can also cause neurotoxicity [26] but these effects may appear first several years after treatment [27]. Furthermore, cisplatin can cause lesions in children through its ototoxicity, and thus, cause damage to the vestibular and auditory systems [28]. The severity of this damage depends on the cumulative dose of the agent received and the duration of treatment. Commonly, Cisplatin causes more damage to the sensory cells in the semicircular canals than in the otolith organs [29], and thus its ototoxicity can result in dizziness, disequilibrium and poor oculomotor control. Ifosfamide is also known to have toxic effects on the CNS. This alkylating agent has well-documented efficacy against a large number of malignant diseases but can cause metabolic encephalopathy of varying severity [30]. Hence, many of the agents commonly used to treat childhood cancer have known side-effects on human sensory and motor functions.

### Subjective symptoms and eye movements

The questionnaire demonstrated that CTS experienced a range of symptoms. Taylor and colleagues [31] showed that common late effects from chemotherapy in childhood included impaired learning and memory (38%) and visual problems (31%). Our questionnaire targeted dizziness and related functions and our subject selection criteria were stricter in order to isolate the effects from chemotherapy. Still, the similarity in self-reported symptoms between studies was striking. We observed concentration problems in 52% and visual disturbance in 70% of CTS. Additionally, we found high rates of headache (96%), unsteadiness, light-headedness or the feeling that things around them were spinning or moving (87%).

Previous reports have illustrated that the self-perception of dizziness, scored by a number-based questionnaire, can accurately predict physiological balance function [32]. This study revealed significant relationships between the subjects’ self-perception of visual disturbances, light-headedness and deficits in oculomotor performance. However, the results in Table 5 also reveal a more complex relationship between many self-reported symptoms and decreased oculomotor performance, where several symptoms stand out as being highly sensitive. Given the diversity of the co-occurring symptoms experienced in CTS, impairments may not be restricted to one function only but may cause long-term impairment to various neurophysiological functions.

To summarize, chemotherapy in childhood or adolescence commonly causes diverse long-term late effects, including impaired oculomotor functions. However, diagnosis of these oculomotor deficits cannot be made by “naked-eye” observation but requires the sensitive clinical eye movement recording and analyses techniques employed by neurology or otology clinics. Since it is not well-known that some chemotherapy treatments given at young age can commonly cause long-term severe late effects, most survivors of childhood cancer are not followed-up for the purpose of preventing, detecting and treating late effects. Moreover, a lack of knowledge about these problems may lead to a delayed development of preventative techniques or therapies.
